# Secreted Rv1768 From RD14 of *Mycobacterium tuberculosis* Activates Macrophages and Induces a Strong IFN-γ-Releasing of CD4^+^ T Cells

**DOI:** 10.3389/fcimb.2019.00341

**Published:** 2019-10-14

**Authors:** Chun-Hui Yuan, Simin Zhang, Feiyan Xiang, Hongjian Gong, Qian Wang, Yan Chen, Wei Luo

**Affiliations:** ^1^Department of Laboratory Medicine, Wuhan Medical and Health Center for Women and Children, Tongji Medical College, Huazhong University of Science and Technology, Wuhan, China; ^2^Department of Emergency, Wuhan Medical and Health Center for Women and Children, Tongji Medical College, Huazhong University of Science and Technology, Wuhan, China; ^3^Clinical Research Center, Wuhan Medical and Health Center for Women and Children, Tongji Medical College, Huazhong University of Science and Technology, Wuhan, China; ^4^Key Research Laboratory for Infectious, Disease Prevention for State Administration of Traditional Chinese Medicine, Department of Pathology, Tianjin Haihe Hospital, Tianjin, China; ^5^Department of Clinical Laboratory, Tianjin Medical University General Hospital, Tianjin, China

**Keywords:** Rv1768, macrophage, tuberculosis, enzyme-linked immunospot, diagnosis

## Abstract

As the first line defensive mediators against *Mycobacterium tuberculosis* (*M.tb*) infection, macrophages can be modulated *by M.tb* to influence innate and adaptive immunity. Recently, we have identified several potential immunodominant T-cell antigens from the region of deletion (RD) of *M.tb* H37Rv, including Rv1768 from RD14. In this study, we further determined that Rv1768 was highly conserved among virulent *M.tb* strains and mainly distributed as a secreted protein. Exposure to recombinant purified Rv1768 (rRv1768) induced apoptosis of bone marrow derived macrophages (BMDMs) but showed no dose-dependent manner. Regarding macrophage activation, significant higher levels of iNOS and pro-inflammatory cytokines (like IL-6 and TNF-α) were detected in rRv1768-challenged BMDMs, whereas arginase 1 (Arg1) expression was markedly decreased. Meanwhile, MHC-II expression and antigen presentation activity of BMDMs were also enhanced by rRv1768 stimulation, leading to significantly increased IFN-γ expression of CD4^+^ T cells isolated from H37Rv-infected mice. It is worthy to note that Rv1768-induced IFN-γ production of peripheral blood mononuclear cells (PBMCs) and Rv1768-specific immunoglobulins was specifically observed in H37Rv-infected mice, but not BCG-infected or normal mice. Analysis of clinical blood samples further revealed that Rv1768 had a higher sensitivity and specificity (91.38 and 96.83%) for tuberculosis diagnosis than the results obtained from clinical CFP10 and ESAT6 peptides (CE)-based enzyme-linked immunospot (ELISPOT) assay. The area under ROC curve of Rv1768 was 0.9618 (95% CI: 0.919–1.000) when cutoff value set as 7 spots. In addition, Rv1768-specific IgG and IgM also exhibited moderate diagnostic performance for tuberculosis compared with CE specific antibodies. Our data suggest that Rv1768 is an antigen that strongly activates macrophages and has potential to serve as a novel ELISPOT-based TB diagnostic agent.

## Introduction

*Mycobacterium tuberculosis* (*M.tb*), is estimated to infect nearly a third of the global population but only a smaller subset of infected individuals present with symptomatic active tuberculosis (TB) (Cadena et al., 2017). This natural variability in outcomes of *M.tb* infection reflects the inherent capacity of the human immune response to control *M.tb* infection but also the fact that this immune control is clearly imperfect (Simmons et al., 2018). As the first-responding mediators against *M.tb* infection, macrophages engulf and eradicate infecting mycobacteria, and also activate adaptive immune responses with the capacities of releasing cytokines or chemokines, as well as antigen presentation (Berg et al., 2016). On the other hand, protective macrophages can also be subverted into a growth-permissive state by different stimuli from *M.tb*, and thus facilitate mycobacterial immune escape (Cronan et al., 2016; Chai et al., 2019). Therefore a more thorough understanding of *M.tb*-macrophage interaction would support the development of new vaccines and anti-TB therapeutic approaches (Cambier et al., 2017).

Through genomics analysis, 129 coding sequences, designated as a region of deletion (RD) proteins, have been found absent from BCG and/or *Mycobacterium bovis* but present in virulent *M.tb* H37Rv (Behr et al., 1999). The most prominent RD protein is ESAT6, which enhances intracellular survival and dissemination of *M.tb* depending on its C-terminal six amino acid residues to inhibit autophagy (Behura et al., 2019), M1 polarization (Refai et al., 2018), and antigen presentation function (Sreejit et al., 2014) of macrophages. Thus, as an widely used immunodominant antigen, ESAT6 was usually combined with other RD proteins (like CFP10, Ag85B, and Rv2660c) for vaccine design (clinical trial identifier: NCT02729571, NCT01865487, NCT02933281) (Aguilo et al., 2017; Suliman et al., 2019) and clinical TB diagnosis with the QuantiFERON-TB (QFT) and T-SPOT assays (Luo et al., 2019). Unfortunately, ESAT6 was only immunogenic in QFT-negative participants (Suliman et al., 2019) and exhibited moderate diagnostic performance in children with *M.tb* infection (Schepers et al., 2014).

Recently, PE/PPE proteins located in RD regions are considered as pivotal candidates for TB vaccine development and diagnostic agents as they have conserved N-terminal Pro-Glu (PE) or Pro-Pro-Glu (PPE) motifs, respectively (Abraham et al., 2018). Rv2352c (also named PPE38), located in RD5, inhibits macrophage MHC-I expression and dampens CD8^+^ T cell responses (Meng et al., 2017). Furthermore, mutation of PPE38 results in the loss of PE_PGRS/PPE-MPTR secretion and links to increased virulence of clinical *M.tb* strains of the Beijing lineage (Ates et al., 2018a). However, introduction of PPE38 to restore PE_PGRS/PPE-MPTR secretion in recombinant BCG neither altered global antigenic presentation or activation of innate immune cells, nor protective efficacy in mouse vaccination-infection models (Ates et al., 2018b). ESAT6, CFP10, Ag85B, and Ag85A are secreted RD proteins (Rodo et al., 2019). And importantly, we have previously confirmed that Rv2645 (RD13) is a secreted antigen, evokes the high level of antigen-specific IFN-γ production and is a potential cell-mediated TB diagnostic agent (Luo et al., 2015). Furthermore, BCG::Rv2645 exhibited enhanced protective efficacy against virulent *M.tb* H37Rv challenge in both mice and rhesus monkeys (Luo et al., 2018). Thus, we speculated that secreted RD proteins lurking in PE/PPE families may have potential capacity to induce protective immune responses and be used as diagnostic antigens.

Previously, we have evaluated the ability of more than 40 RD proteins, mainly from RD1-3 and RD10-14 segments of *M.tb* H37Rv, to induce specific IFN-γ production by ELISPOT and have found that Rv1768 (PE_PGRS31) evoked the higher TB-specific IFN-γ, response ranked only second to Rv2645 (Luo et al., 2015). In this study, we further evaluated the properties and immune characteristics of Rv1768 and confirmed that Rv1768 was highly conserved among virulent *M.tb* strains and mainly distributed as a secreted protein. Molecular studies revealed that recombinant Rv1768 (rRv1768) promoted M1 polarization and enhances the antigen presentation ability of BMDMs *in vitro*. More importantly, Rv1768-induced IFN-γ production of peripheral blood mononuclear cells (PBMCs) and Rv1768-specific immunoglobulins (IgG and IgM) were only observed in H37Rv-infected mice, but absent in BCG-infected or normal mice. Based on these promising results, we further tested the diagnostic potential of Rv1768 in clinical samples. As expected, Rv1768 specifically induced a high level of IFN-γ production in PBMC of active TB patients and displayed better immunoreactivity than CFP-10 and ESAT6 peptides (CE). In addition, Rv1768-specific immunoglobulins also exhibited moderate diagnostic performance for tuberculosis.

## Materials and Methods

### Participants

A total of 121 participants were enrolled in the study, including 63 patients with active pulmonary TB and 58 healthy controls. Sixty three PBMC samples (for both clinical CE-based T-SPOT.TB and Rv1768-based ELISPOT) and 45 serum samples (for antibody test) from TB were collected from Tianjin Haihe Hospital. Fifty eight PBMC samples and 45 serum samples from healthy controls (HCs) were obtained from Tianjin Medical University General Hospital and Wuhan Medical and Health Center for Women and Children. Enrollment criteria of all subjects was the same as we previously described (Luo et al., 2015). In brief, subjects were enrolled and divided into different groups according to the following criteria: (1) clinical signs or symptoms of TB; (2) TB contact history; and (3) chest X-ray. The study was approved by the ethics committee of Tianjin Medical University General Hospital, and Tianjin Haihe Hospital. Written informed consent was obtained from all participants.

### Bacterial Strains and Animal Protocol

*M.tb* H37Rv [strain ATCC 25618] and *M. bovis* BCG [strain ATCC 35734] were maintained on 7H9 middle brook liquid medium supplemented with 10% oleic acid-albumin-dextrose-catalase (OADC) (BD Difco, USA) and harvested while in log-phase growth. Bacilli were washed in phosphate buffered saline (PBS) with 0.05% Tween 80 and triturated uniformly before use (Yuan et al., 2019).

BALB/c mice (6–8 weeks of age) obtained from Animal Laboratory Center of Wuhan University were used in this study. The mice were *i.v*. infected with BCG or *M.tb* H37Rv (1 × 10^5^ CFU/mouse) in the ABSL-3 Laboratory of the Wuhan University School of Medicine (Luo et al., 2018; Yuan et al., 2019). After 30 days of infection, mice were sacrificed by carbon dioxide inhalation. Serum and splenocytes were then harvested. The animal experimental protocol was approved by the Institutional Animal Care and Use Committee of Wuhan University.

### Recombinant Rv1768 (rRv1768) Protein Preparation

The Rv1768 gene sequence has been amplified by PCR from the genomic DNA of *M.tb* H37Rv and subcloned into the 6 × His-tagged expression vector pET28a as we previously described (Luo et al., 2015). The recombinant Rv1768-pET28a vector was then transformed into *Escherichia coli* (*E. coli*) BL21 (DE3) and treated with 50 mM Isopropyl-β-D-thiogalactoside (IPTG, Sigma-Aldrich) for 16 h at 25°C, inducing rRv1768 protein expression. rRv1768 was purified by immobilized metal affinity chromatography (IMAC) according to the manufacturer's protocol (QIAGEN, GERMAN) and confirmed by SDS-PAGE or western blot. Endotoxin in the purified protein was removed using polymyxin affinity chromatography (Bio-Rad, Shanghai, China). Protein concentration of rRv1768 was determined by the bicinchoninic acid method.

### Western Blot

BL21 harboring rRv1768-pET28a plasmid was induced by IPTG for different time periods (6, 12, 24, and 48 h), and then the supernatant and cell pellets were harvested by centrifuging at 12,000 g for 5 min. The proteins were subjected to SDS-PAGE and transferred to PVDF membrane. The membrane was blocked with 5% non-fat milk at 4°C overnight, followed by incubation with His-tag Mouse mAb (1:2000, Sungene, China) at 37° C for 1 h. After washing with TBST three times, the membrane was then incubated with horseradish peroxidase (HRP) conjugated goat anti-mouse IgG (1:3,000, Affinity, USA). CFP10 and Rv1773 were served as secreted and intracellular protein control, respectively, as we previously described (Luo et al., 2015).

To determine iNOS and Arg-1 expression in Bone Marrow Derived Macrophages (BMDM), BMDM (2 × 10^6^/mL) were stimulated with rRv1768 (5 μg/ml) for 24 h. Then, cells were lysed and subjected to SDS-PAGE. Anti-iNOS mAb was obtained from Abcam (Cambridge, U.K.) and Anti-Arg-1 mAb was purchased from Cell Signaling (Beverly, MA, USA) (Sun et al., 2016). Chemiluminescent detection was performed by using ECL Plus Western blotting reagents.

### *In vitro* Differentiation of BMDM

BMDMs were prepared as we previously described (Tang et al., 2017). Briefly, bone marrow cells isolated from the femurs and tibias of BALB/c mice were treated with ACK Lysis Buffer (Beyotime, Shanghai, China) and cultured in DMEM (Gibco, UK) supplemented with 10% FBS (Gibco), 1% Penicillin-Streptomycin Solution, and 50 ng/ml macrophage colony stimulating factor (M-CSF, Peprotech) for 6 days to induce differentiation into BMDM (the adherent cells). The purity of BMDM (F4/80^+^) was determined by flowcytometry (FCM).

### Flowcytometry (FCM)

Apoptosis of BMDM were determined by using FITC Annexin V apoptosis detection kit with Propidium Iodide (PI) (BD biosciences), MHC-II expression of BMDM in response to rRv1768 stimulation was measured by PerCP/Cy5.5 anti-MHC-II (Biolegend, CA, USA). Cytokines production in culture supernatant of rRv1768 stimulated BMDM was determined using mouse Th1/Th2/Th17 cytometric-beads array kit (BD PharMingen, USA), according to the manufacturer's protocol. Intracellular IFN-γ expression of CD3^+^CD4^+^ T cells was determined by staining with APC-anti-CD3, FITC-anti-CD4, PE-anti-IFN-γ (Biolegend, CA, USA). All procedures were performed as we previously described (Tang et al., 2017; Yuan et al., 2019) and analyzed on BD Accuri C6 Flow cytometer (BD Biosciences).

### Magnetic Activated Cell Sorting (MACS)

The EasySep™ Mouse CD4^+^ T Cell Isolation Kit (Stemcell Technologies, Canada) was used to isolate CD4^+^ T cells from splenocytes of H37Rv infected BALB/c mice by negative selection. The labeled cells were separated using an EasySep™ magnet, and the desired CD4^+^ T cells were poured off into a new tube. The purity of CD4^+^ T cells was >95% as determined by FCM.

### Antigen Processing and Presentation

BMDM (5 × 10^5^ cells/well) were stimulated with rRv1768 protein (5 μg/mL) or control buffer in the presence of IFN-γ (15 ng/mL) for 24 h at 37°C. BMDM cells were then washed and incubated with heat inactivated *M.tb* H37Rv (MOI = 2) for 3 h. After being fixed in 1% paraformaldehyde and washed extensively, BMDM cells were finally co-cultured with CD4^+^ T cells isolated from splenocytes of H37Rv infected BALB/c mice (1 × 10^6^/well) for 48 h. Intracellular IFN-γ expression of CD4^+^ T cells was determined by FCM.

### Enzyme-linked Immunospot (ELISPOT) Assay

Rv1768-specific IFN-γ production of mouse splenocytes was determined by mouse ELISPOT IFN-γ assay (DAKEWE Biotechnology, Beijing, China), as we previously described (Luo et al., 2015). Briefly, 2.5 × 10^5^ splenocytes from normal, BCG or H37Rv infected mice were stimulated with rRv1768 protein (5 μg/ mL) per well in EZ-Culture^TM^ serum-free culture medium (DAKEWE Biotechnology, Beijing, China) for 24 h, and then added into the mouse anti-IFN-γ monoclonal antibody (mAb) precoated plate. PHA and PBS were used as a positive and negative control, respectively. Human Rv1768-specific IFN-γ production of TB patients and HCs in response to Rv1768 stimulation, and clinical CE-based T-SPOT.TB were determined as we previously described (Luo et al., 2015). Responses were measured by counting the spot-forming unit (SFU). All samples were tested in duplicate wells and all data are from three independent experiments.

### Serum Antibody Measurement

Serum Rv1768 and CE protein-specific IgG or IgM were determined as we previously described (Luo et al., 2015). Briefly, 96-well microtiter plates were coated with individual rRv1768 proteins and CE fusion protein, respectively, at 10 μg/ mL (100 μL/well) in coating buffer (0.1 M carbonate/bicarbonate, pH 9.6) and incubated at 4°C overnight. After washing four times with PBS containing 0.05% (v/v) Tween-20 (PBST), the plates were blocked with 200 μl/well blocking buffer [2% bovine serum albumin (BSA) in PBST] at 37°C for 1 h. After washing, 200-fold diluted human serum samples were added and incubated at 37°C for 1 h. The plates were thoroughly washed and then incubated with HRP-conjugated goat anti-human IgG and IgM antibodies at 37°C for 30 min, followed by the addition 100 μL/well of TMB substrate. The reaction was stopped by the addition of 50 μl of 2 M H_2_SO_4_. The optical densities were then measured at 450 nm within 10 min with a PerkinElmer 2030 multilabel reader. All samples were tested in duplicate wells in each experiment and all data were from three independent experiments.

### Statistical Analysis

Data are presented as mean ± SD and analyzed by GraphPad Prism V 6.00 (GraphPad Software, San Diego, CA). Statistical significance was determined by Student *t*-test or ANOVA followed by Neuman-Keuls *post hoc* test. A *P* < 0.05 was considered as statistically significant (^*^P < 0.05, ^**^P < 0.01, ^***^P < 0.001). Diagnostic performance was examined by analysis of receiver operating characteristic (ROC) curves, and the area under the curve (AUC) was calculated accordingly. Sensitivity was determined by dividing the number of positive cases by the total number of TB patients. Specificity was determined by dividing the number of negative controls by the total number of HCs. The cut-off OD value was determined using Youden's index (YI). YI was calculated (YI = sensitivity + specificity – 1) for each coordinate point of the ROC curve to determine the cut-off value, which has the maximum sensitivity and specificity pair.

## Results

### Rv1768 Is a New Type of Early Secreted RD14 Protein

**Figure 1:**
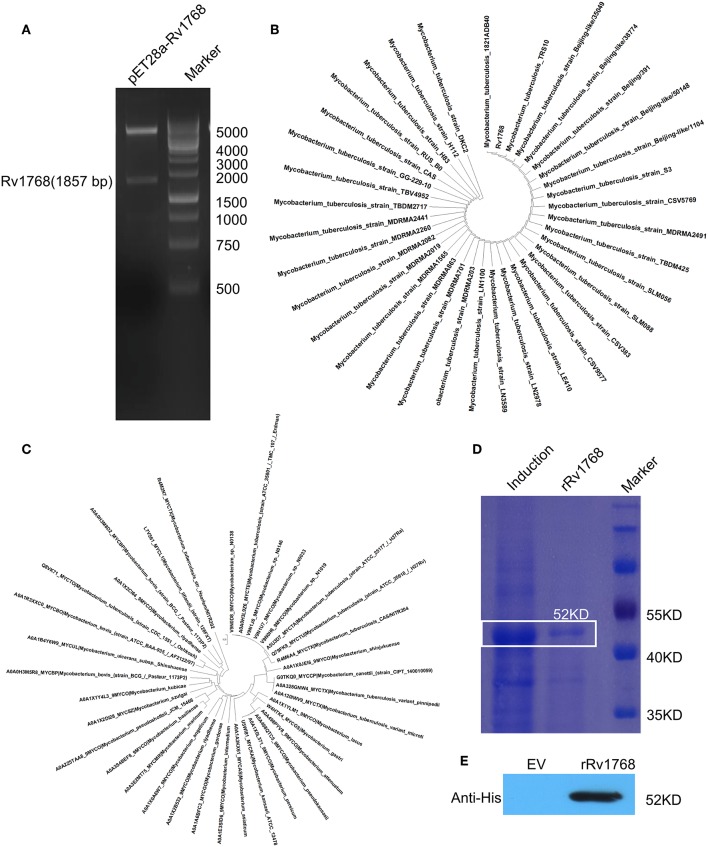
Expression and purification of Rv1768. **(A)** Double enzyme digestion of recombinant plasmid pET28a-Rv1768. **(B)** Phylogenetic analysis of Rv1768 CDS sequence in mycobacterial species acquired by blasting of *M.tb* Rv1768 (https://mycobrowser.epfl.ch/genes/Rv1768) against NCBI database. **(C)** Phylogenetic analysis of Rv1768 homologs in mycobacterial species acquired by blasting of *M.tb* Rv1768 (Q79FK9_MYCTU) against UniProtKB_Bacteria database. **(D)** Purification and SDS-PAGE of Rv1768. **(E)** Western blot identification of Rv1768 with anti-His mAb, empty vector (EV) was used as a control.

As described in our previously study (Luo et al., 2015), we have cloned more than 40 different RD genes of *M.tb* H37Rv into pET28a, including Rv1768 (Figure 1A). BLAST search in NCBI and UniProtKB_Bacteria database, followed by phylogenetic analysis, indicated that Rv1768 DNA sequence (Figure 1B) and protein homologs (Figure 1C) were only present in mycobacterial species and highly conserved among the pathogenic mycobacteria, including isolated clinical Beijing sub-strains. Rv1768-His-tag recombinant protein (rRv1768) was then purified and identified by SDS-PAGE (Figure 1D) and Western blot (Figure 1E). rRv1768 evoked higher numbers of IFN-γ-expressing cells among all of the tested RD proteins, including the well-known RD1 antigens CFP10, ESAT6, and Ag85B, and only lower than Rv2645 (Yuan et al., 2019).

**Figure 2:**
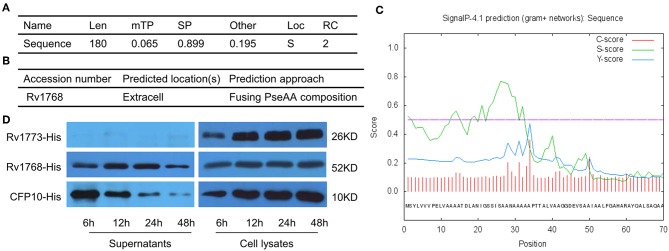
The secreted prediction of Rv1768. **(A)** The prediction of the subcellular localization of Rv1768 protein. **(B)** Gpos-PLoc: predicting Gram-positive bacteria protein subcellular location. **(C)** The signal peptide prediction of Rv1768 protein. **(D)** The supernatants of BL21-Rv1768 and whole cell lysates analyzed by Western blot with anti-His mAb, CFP10, and Rv1773 were served as secreted and intracellular protein control, respectively.

To further evaluate the characteristics of Rv1768, its amino acid sequence was obtained from the uniprot (https://www.uniprot.org/uniprot/) and then subjected to TargetP 1.1 Server (http://www.cbs.dtu.dk/services/TargetP/) for prediction of subcellular localization. The possibility that Rv1768 protein belongs to the secreted protein is 0.899, and the probability in other cellular positions is 0.195 (Figure 2A), In addition, the result of Gpos-PLoc (http://www.csbio.sjtu.edu.cn/bioinf/Gpos-multi/) also confirmed that Rv1768 is an extracellular protein of *M.tb* (Figure 2B). We then sed further used SignalP 4.1 Server (http://www.cbs.dtu.dk/services/SignalP/) to predict whether Rv1768 has signal peptide, and it was found that the D value of Rv1768 was 0.493 > cut off value of 0.450 (Figure 2C), which indicated that a signal peptide was found in Rv1768 amino acid sequence. In order to confirm the cellular location of Rv1768, we then collected supernatants and whole cell lysates of BL21-Rv1768 at indicated time points (6, 12, 24, and 48 h post IPTG induction). Western blot analysis revealed that Rv1768, in accordance with CFP10 and Rv1773, has high content in the cell lysates at each time point (Figure 2D). Similarly, to secreted protein CFP10, Rv1768 was secreted into the supernatants as early as 6 h and this continued up until 48 h (Figure 2D). As a transcriptional repressor deleted from BCG-Pasteur^27^, Rv1773 protein was not detected in the supernatant (Figure 2D). Thus, these results indicated that Rv1768 is a secreted protein of *M.tb*.

### Rv1768 Enhances M1 Polarization and Antigen Presentation of Macrophages

**Figure 3:**
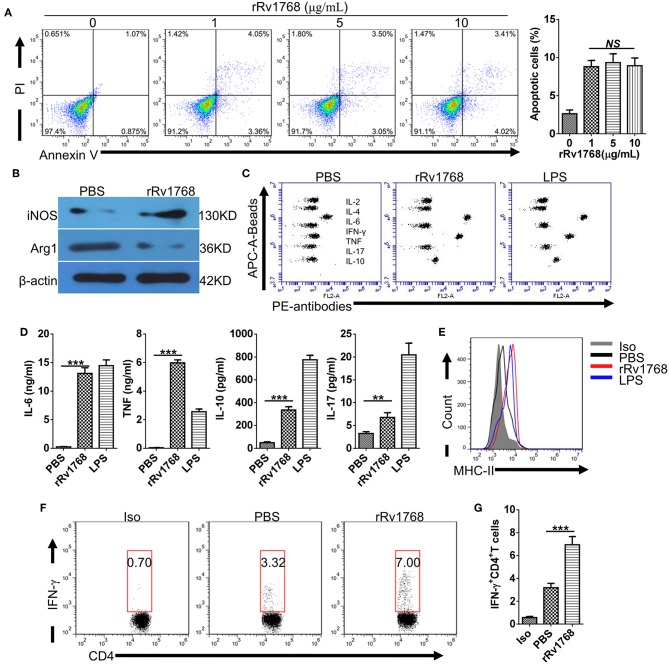
Rv1768 promotes M1 polarization and antigen presentation activity of BMDMs. **(A)** BMDMs were treated with rRv1768 at indicated concentrations for 24 h and then apoptosis was detected by FCM. **(B)** The expression of iNOS and Arg1 of BMDMs were determined by Western Blot. **(C)** Cytokines production of BMDMs were detected by CBA method. **(D)** Production of different cytokines in **(C)** were presented. **(E)** BMDMs were stimulated with or without rRv1768 (5 μg/mL), the expression of MHC-II was analyzed by FCM. **(F)** BMDM (5 × 10^5^ cells/well) were stimulated with rRv1768 protein (5 μg/ml) or control buffer in the presence of IFN-γ (15 ng/ml) for 24 h at 37°C. Then, BMDM cells were washed and incubated with heat inactivated *M.tb* H37Rv (MOI = 2) for 3 h. After fixed in 1% paraformaldehyde and washed extensively, BMDM cells were finally co-cultured with CD4^+^ T cells isolated from splenocytes of H37Rv infected mice (1 × 10^6^/well) for 48 h. Intracellular IFN-γ expression of CD4^+^ T cells was determined by FCM. Iso, Isotype of IFN-γ antibody. **(G)** Percentage of IFN-γ^+^CD4^+^ T cells in **(F)** were presented. The data are shown as mean ± SD of four independent experiments performed in triplicates. **P < 0.01, ***P < 0.001.

Considering the release of Rv1768 in the culture supernatant of *M.tb*, we further evaluated the biological function of Rv1768 on macrophages. BMDMs were stimulated with increasing concentration (0–10 μg/ml) of rRv1768 for 24 h, and then cellular apoptosis was measured by FCM. As shown in Figure 3A, Rv1768 induced apoptosis of BMDMs but demonstrated no dose-dependent manner. We then measured polarization and cytokines production of BMDMs in the context of 5 μg/ml rRv1768 stimulation. Rv1768 significantly enhanced iNOS and inhibited Arg1 expression in BMDMs (Figure 3B). In addition, CBA assay showed that IL-6 and TNF production were significantly increased in rRv1768-treated BMDMs (Figures 3C,D). Thus, these results suggest that Rv1768 promotes BMDMs toward M1 polarization.

Prolonged incubation with mycobacterial antigens modulates MHC-II expression and antigen-presentation activity of macrophages (Su et al., 2016). To determine whether Rv1768 regulates MHC-II expression, BMDMs were stimulated with or without rRv1768 for 24 h. As shown in Figure 3E, Rv1768 stimulation enhanced MHC-II expression of BMDMs. After pulsing with inactivated H37Rv and then co-cultured with CD4^+^ T cells isolated from H37Rv infected mice, we further found that Rv1768 enhanced the frequency of IFN-γ-expressing CD4^+^ T cells (6.95% ± 1.23 vs. 3.19% ± 0.65 in PBS group) (Figures 3F,G). IFN-γ is a pivotal effector for controlling *M.tb* infection and is a diagnostic indicator for TB (Su et al., 2017). These findings suggest that Rv1768 is an activator of MHC-II expression and antigen-presentation and may have potential to be used as a diagnostic agent.

### Rv1768-indcued IFN-γ and Antibodies Specifically Correlated With *M.tb* Infection

**Figure 4:**
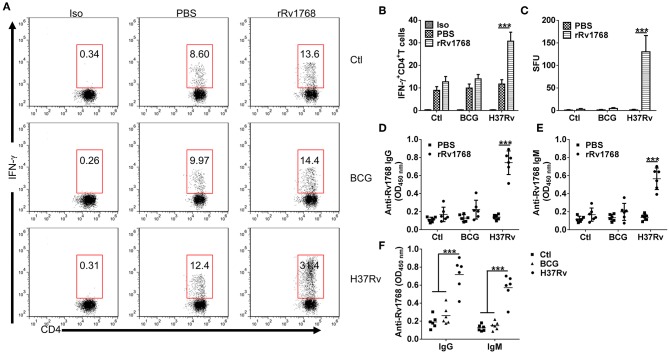
Rv1768 specifically stimulates IFN-γ production and Rv1768-specific Igs in splenocytes of H37Rv-infected mice. **(A)** Splenocytes isolated from normal control, BCG, and H37Rv infected mice were stimulated with rRv1768 (5 μg/ml) for 24 h, intracellular IFN-γ expression of CD4^+^ T cells was determined by FCM. Iso, Isotype of IFN-γ antibody. **(B)** Percentage of IFN-γ^+^CD4^+^ T cells in **(A)** were presented, and the data are shown as mean ± SD of six mice per group performed in triplicates, ***P < 0.001. **(C)** Same as **(A)**, Rv1768-specific IFN-γ production of mouse splenocytes was determined by mouse ELISPOT IFN-γ assay. The data were presented as mean ± SD of six mice per group performed in triplicates, ***P < 0.001. Splenocytes isolated from normal control, BCG and H37Rv infected mice were stimulated with rRv1768 (5 μg/ml) for 72 h, Rv1768-specific IgG **(D)** and IgM **(E)** in supernatant were determined by ELISA. The data were presented as mean ± SD of six mice per group performed in triplicates, ***P < 0.001. **(F)** Serum Rv1768-specific IgG and IgM in normal control, BCG and H37Rv infected mice were determined by ELISA. The data were presented as mean ± SD of six mice per group performed in triplicates, ***P < 0.001.

We previously found that Rv1768 evoked higher numbers of IFN-γ-expressing cells in splenocytes from inactivated H37Rv-immunized mice (Luo et al., 2015). To confirm whether Rv1768-evoked IFN-γ specifically correlated with *M.tb* infection. Splenocytes from normal and BCG or H37Rv infected mice (Supplemental Figures 1A,B) were stimulated with Rv1768 and then IFN-γ-expressing CD4^+^ T cells were determined by FCM. Rv1768 specifically increased frequency of IFN-γ-expressing CD4^+^ T cells in H37Rv infected mice (11.75% ± 1.89 vs. 30.75% ± 3.96) and had no significant impact on CD4^+^ T cells isolated from normal and BCG infected mice (Figures 4A,B). In addition, ELISPOT assay also confirmed that Rv1768 specifically enhanced frequency of IFN-γ-expressing cells in H37Rv infected mice (Figure 4C, Supplemental Figure 1C). As Rv1768 enhances antigen-presentation activity of macrophages, we then further determined Rv1768 specific antibodies in supernatant of splenocytes. As shown in Figures 4D,E, IgG and IgM targeted to Rv1768 were significantly increased in Rv1768-pulsed splenocytes isolated from H37Rv infected mice, while they no obvious change in normal or BCG infected mice. Similar results were also obtained in serum of normal and BCG or H37Rv infected mice (Figure 4F). Thus, these results demonstrate that Rv1768-induced IFN-γ and antibodies specifically correlated with *M.tb* infection.

### Rv1768-specific IFN-γ Levels Was Higher in Active TB Patients Than in HCs

**Figure 5:**
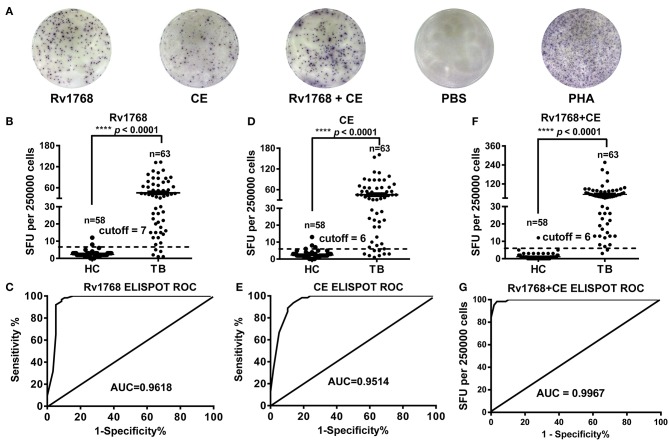
Rv1768, CE, and Rv1768 plus CE specific IFN-γ levels are much higher in active tuberculosis patients than in healthy controls. **(A)** Representative IFN-γ spots morphologies of Rv1768 induced PBMCs responses in active tuberculosis (TB) patients and healthy controls (HC). **(B)** Comparison of Rv1768 specific IFN-γ levels between TB patients and HCs. **(C)** ROC curve of Rv1768-based ELISPOT. **(D)** Comparison of CE specific IFN-γ levels between TB patients and HCs. **(E)** ROC curve of CE-based ELISPOT. **(F)** Comparison of Rv1768 plus CE specific IFN-γ levels between TB patients and HCs. **(G)** ROC curve of Rv1768 plus CE-based ELISPOT.

In order to confirm whether Rv1768-induced IFN-γ can be used for TB diagnosis, we isolated PBMCs from 58 healthy controls and 63 active TB patients and then stimulated with Rv1768 protein. The clinical characteristics of the study populations are described in Supplemental Table 1. ELISPOT was used to determine the number of IFN-γ-expressing cells (Figure 5A). Our results revealed that Rv1768 can induce higher numbers of IFN-γ-expressing cells in active TB patients compared with healthy controls (Figure 5B). Furthermore, the AUC of Rv1768 ELISPOT reached 0.9618 (95% CI: 0.9191–1.0000, cutoff value: 7), with high sensitivity (91.38%) and specificity (96.83%) (Figure 5C), which was better than the AUC of CE 0.9514 (95% CI: 0.9124–0.9904, cutoff value: 6) with sensitivity and specificity of 86.21 and 93.65%, respectively (Figures 5D,E). When combined Rv1768 with CFP10-ESAT6 (Rv1768 + CE), the AUC reached 0.9967 (95% CI: 0.9914–1.0000, cutoff value: 6), with high sensitivity and specificity of 96.55 and 98.41%, respectively (Figures 5F,G). These results indicated that TB individuals release high IFN-γ levels against Rv1768, and Rv1768 is also sensitive for the diagnosis of TB individuals from healthy controls.

### Rv1768 Protein Induces Stronger Antibody Responses in TB as Compared to CE

**Figure 6:**
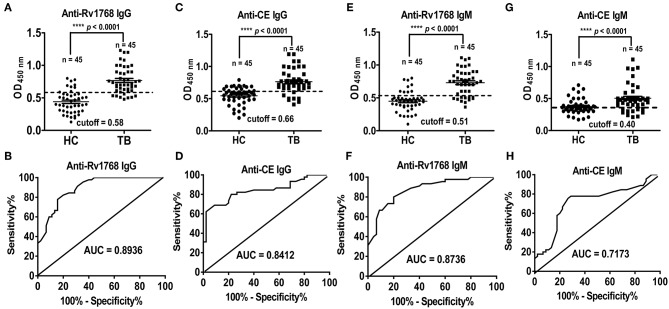
Rv1768 elicits stronger antibody responses in active TB compared to CE group. **(A)** Level of Rv1768-specific IgG in TB patients and HCs. **(B)** The ROC curve of Rv1768-specific IgG. **(C)** Level of CE-specific IgG in TB patients and HCs. **(D)** The ROC curve of CE-specific IgG. **(E)** Level of Rv1768-specific IgM in TB patients and HCs. **(F)** The ROC curve of Rv1768-specific IgM. **(G)** Level of CE-specific IgM in TB patients and HCs. **(H)** The ROC curve of CE-specific IgM.

We previously found that serological antibody actually plays a dominant role in the diagnosis of TB patients with higher detection rate, sensitivity and specificity (Luo et al., 2015). In this study, we observed that the expression of IgG and IgM induced by Rv1768 in TB patients are preceded than which in HCs, the detection of CE protein specific serological antibody as a positive control. The ROC curve area AUC of Rv1768-IgG can reach 0.8936 (95% CI: 0.8299–0.9572), with high sensitivity and specificity of 82.22 and 80.00%, respectively, and a cutoff value of 0.58 (Figures 6A,B). This was better than CE-IgG, which can reach 0.8412 (95% CI: 0.7567–0.9258) with a high sensitivity and specificity of 80% and 77.78%, and a cutoff value of 0.66 (Figures 6C,D). Similarly to IgG, the ROC curve area AUC of Rv1768-IgM can reach 0.8736 (95% CI: 0.8022–0.9450), with a high sensitivity and specificity of 77.78 and 80.00%, respectively, and a cutoff value of 0.51 (Figures 6E,F). This is better than CE-IgM, which can reach 0.7173 (95% CI: 0.6071–0.8275), with a high sensitivity and specificity of 75.56 and 73.33%, respectively, and cutoff value of 0.40 (Figures 6G,H). The above results suggest that Rv1768-specific serological antibody can also apply to an important auxiliary detection target for the diagnosis of TB.

## Discussion

As the central defensive mediators of the immune response, macrophages can be transformed into a growth-permissive state in response to different stimuli of *M.tb*, like Mannose-capped lipoarabinomannan (ManLAM) and phenolic glycolipid (PGL) (Cambier et al., 2014, 2017). Previously, we have confirmed that ManLAM promotes apoptosis, M2 polarization and inhibits antigen-presenting activity of macrophages (Sun et al., 2016; Pan et al., 2017; Tang et al., 2017). In line with these work, DNA aptamer targeting to ManLAM has a strong potential for use as an immune enhancer of BCG in mice and monkeys models, and clinical findings also revealed the potential of ManLAM for TB diagnosis (Sun et al., 2016; Tang et al., 2016). However, the potential of *M.tb*-derived pathogen-activated molecular patterns (PAMPs) to enhance the anti-microbial function of macrophages is still less well-reported.

In this study, we evaluated the effects of Rv1768 on macrophages following research of a previous study (Luo et al., 2015). Our results showed that Rv1768 induces M1 polarization, production of pro-inflammatory cytokines and promotes antigen presentation activity of macrophages. Rv1768 (PE_PGRS31) locates in RD14 and is a member of the PE_PGRS family (Bachhawat and Singh, 2007). Molecular analysis confirmed that Rv1768 is a secreted antigen and is highly conserved among *M.tb* strains, including isolated clinical Beijing substrains. Fifty six of the sixty one PE_PGRS proteins, including Rv1768, all contain multiple calcium-binding and glycine-rich sequence motifs GGXGXD/NXUX (Yeruva et al., 2016). Among these 56 proteins, PE_PGRS33 (Rv1818c) triggers macrophage cell death and mediates entry of *M.tb* into macrophages through directly interacting with TLR2 in a Ca^2+^-dependent manner (Palucci et al., 2016). However, PE_PGRS29 (Rv1468c) recruits autophagy receptor p62 to deliver *M.tb* into LC3-associated autophagosomes, and thus enhances xenophagic clearance of *M.tb* in macrophages (Chai et al., 2019). To date, we firstly demonstrate that Rv1768 positively enhances the anti-microbial functions of macrophages and exhibits a minor impact on cellular apoptosis, the detailed molecular mechanisms by which Rv1768 targets to need to be further investigated in future.

With the absence of MVA85A efficacy against TB in infants previously vaccinated with BCG (phase 2b trial, NCT00953927) (Tameris et al., 2013) and the moderate performance of current TB diagnostic methods in children, like the established clinical QFT and T-SPOT assays, which contain MTB-specific antigens, ESAT6 and CFP10, encoded by the RD1 region (Luo et al., 2019). Searching for novel immunodominant antigens of *M.tb* that play critical roles in regulating protective immune response is urgently required. PE/PPE proteins located in RD regions are considered to be the most promising candidates for TB vaccine development and diagnostic agents, especially secreted antigens (Abraham et al., 2018). Rv3425 (PPE57), which locates in the RD11 region and induces macrophage activation by augmenting the expression of MHC-II and pro-inflammatory cytokines (TNF-α, IL-6, and IL-12p40), has been confirmed with the potential to distinguish patients with active TB via QFT assay (Wang et al., 2013; Losi et al., 2016) and for use as an antigen for therapeutic or protective vaccine design (Xu et al., 2014; Yang et al., 2015). In this study, Rv1768 evoked a significantly high level of IFN-γ in H37Rv-infected mice, but not BCG-infected or normal mice. Furthermore, Rv1768-based ELISPOT distinguish patients with active TB from BCG-vaccinated individuals. The addition of novel antigens to the QFT assay can strongly help in the development of a new generation of QFT assay with the potential to increase specificity (Losi et al., 2016). In this study, the combination of Rv1768 and CE peptides showed better diagnostic performance compared to CFP10-ESAT6 peptides alone as sensitivity and specificity were increased. Thus, Rv1768 has the potential to be used as a supplemental antigen for the CE-based T-SPOT in a diagnostic peptides' mixture.

Antibodies are powerful biomarkers and important immune mediators during *M.tb* infection, and specifically, antibodies targeting to specific antigens correlate with the disease state of TB (Lu et al., 2016; Zimmermann et al., 2016). Rv1768 specific antibodies significantly increased in Rv1768-pulsed splenocytes of H37Rv-infected mice than in normal or BCG-infected mice. In clinical samples, Rv1768-specific IgG and IgM levels in active TB patients are higher than in Healthy controls, and the ROC curve area AUC of Rv1768-specific antibodies are better than CE. However, the diagnostic sensitivity of Rv1768-specific IgG (82.22%) is less than PPE17 (about 95%) (Abraham et al., 2018). Detection of PPE17-specific IgG also discriminates individuals with latent TB from the QFT-negative subjects and exhibits a higher sensitivity (about 87%), as compared to currently used TB diagnostic antigens like ESAT6, CFP10, and PPD (Abraham et al., 2018). These results indicate that Rv1768 antibodies only exhibit moderate diagnostic performance for TB diagnosis.

In conclusion, our study firstly demonstrated that Rv1768 (PE_PGRS31) is a secreted RD protein, promotes M1 polarization and enhances antigen presentation activity of macrophages. As a highly conserved protein in virulent *M.tb* strains, Rv1768-evoked IFN-γ specifically increased in PBMCs of active TB patients. Furthermore, combination of Rv1768 and CE peptides showed better diagnostic performance compared to CFP10-ESAT6 peptides alone. In this direction, our study provides evidence that Rv1768 may be a novel sero-diagnostic marker for detection of TB and may be used as a supplemental antigen for CFP-10 and ESAT6 in the clinical T-SPOT assay.

## Data Availability Statement

All datasets generated for this study are included in the manuscript/Supplementary Files.

## Ethics Statement

The studies involving human participants were reviewed and approved by the ethics committee of Tianjin Medical University General Hospital, and Tianjin Haihe Hospital. The patients/participants provided their written informed consent to participate in this study. The animal study was reviewed and approved by the ethics committee of Tianjin Medical University General Hospital, and Tianjin Haihe Hospital.

## Author Contributions

WL and C-HY designed the research. YC, SZ, and HG conducted the experiments. QW, WL, and FX collected clinical samples. C-HY, YC, and HG analyzed the data. C-HY and YC wrote the manuscript. WL supervised the research and revised the manuscript.

### Conflict of Interest

The authors declare that the research was conducted in the absence of any commercial or financial relationships that could be construed as a potential conflict of interest.

## Supplementary Material

Supplemental Figure 1 Rv1768 specifically stimulates IFN-γ releasing in splenocytes of H37Rv-infected mice. **(A)** HE stain of lungs obtained from normal control, BCG, and H37Rv-infected mice. **(B)** Acid-fast stain analyses of lung tissue. **(C)** Splenocytes isolated from normal control, BCG, and H37Rv infected mice were stimulated with rRv1768 (5 μg/ml) for 24 h, Rv1768-specific IFN-γ production of mouse splenocytes was determined by mouse ELISPOT IFN-γ assay.

Supplemental Table 1 Clinical characteristics of study participants by classification group.
