# Supplementary figures and images for: Secreted Rv1768 From RD14 of Mycobacterium tuberculosis Activates Macrophages and Induces a Strong IFN-γ-Releasing of CD4+ T Cells
Source: Front Cell Infect Microbiol. 2019 Oct 14;9:341. doi: 10.3389/fcimb.2019.00341 (PMC6802416; doi:10.3389/fcimb.2019.00341)

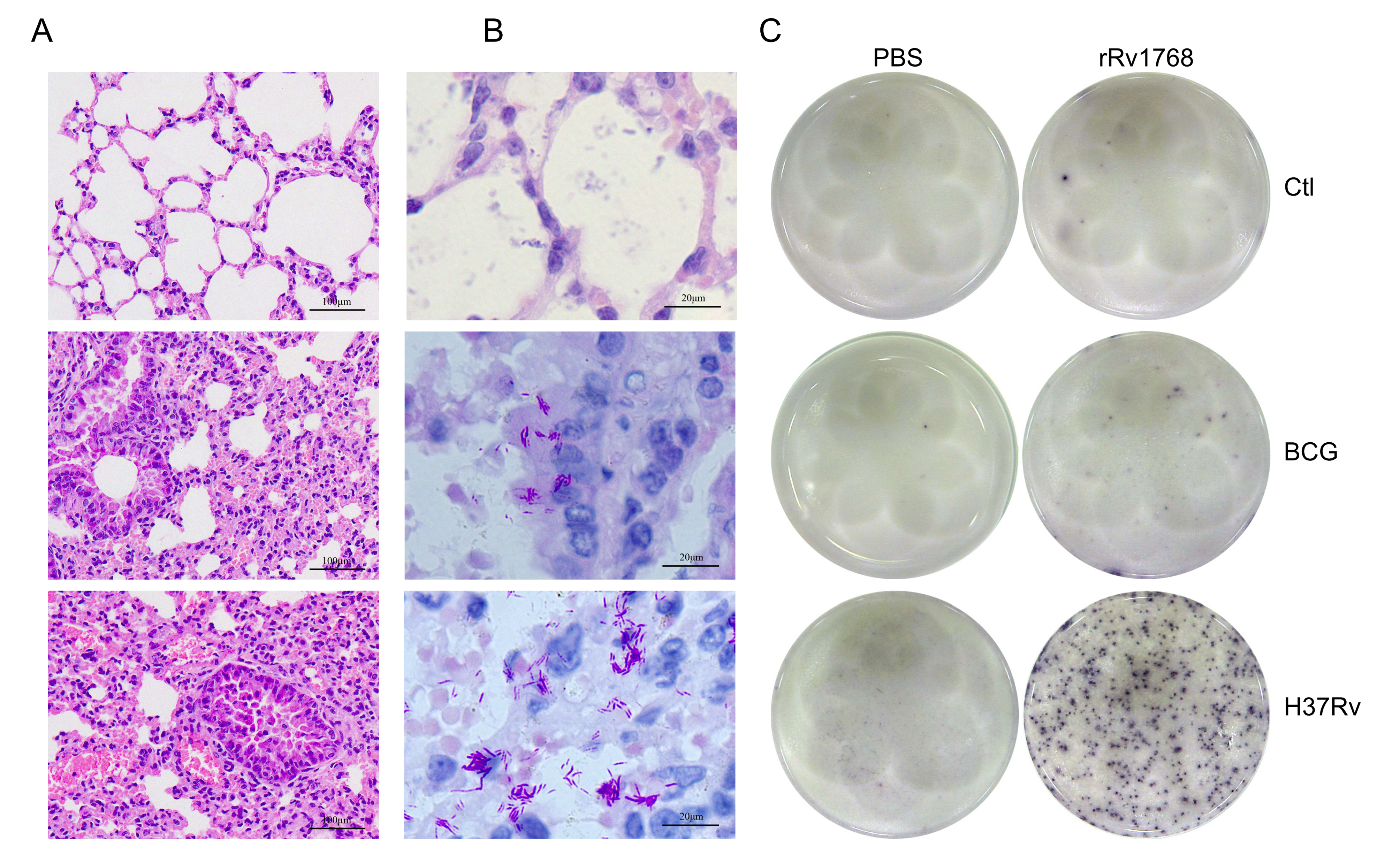

Supplement: Supplemental Figure 1 — Rv1768 specifically stimulates IFN-γ releasing in splenocytes of H37Rv-infected mice. (A) HE stain of lungs obtained from normal control, BCG, and H37Rv-infected mice. (B) Acid-fast stain analyses of lung tissue. (C) Splenocytes isolated from normal control, BCG, and H37Rv infected mice were stimulated with rRv1768 (5 μg/ml) for 24 h, Rv1768-specific IFN-γ production of mouse splenocytes was determined by mouse ELISPOT IFN-γ assay. [file Image_1.TIF]
