# Supplementary material for: Secreted Rv1768 From RD14 of Mycobacterium tuberculosis Activates Macrophages and Induces a Strong IFN-γ-Releasing of CD4+ T Cells
Source: Front Cell Infect Microbiol. 2019 Oct 14;9:341. doi: 10.3389/fcimb.2019.00341 (PMC6802416; doi:10.3389/fcimb.2019.00341)
Supplement: Supplemental Table 1 — Clinical characteristics of study participants by classification group. [file Table_1.DOC]

**Supplemental Table 1. Clinical characteristics of study participants by classification group**

| **Characteristic** | **TB patients for ELISPOT**  **(n = 63)** | **HCs for ELISPOT**  **(n= 58)** | **TB patients for Ab assay**  **(n = 45)** | **HCs for Ab assay**  **(n= 45)** |
| --- | --- | --- | --- | --- |
| **Age (years)** | 45.80 ± 12.36 | 41.57 ± 10.15 | 40.37 ± 16.11 | 39.44 ± 12.27 |
| **Female gender** | 26/63 (41.27%) | 27/58 (46.55%) | 22/45 (48.89%) | 26/45 (57.78%) |
| **Comorbidities** | 4/63 (6.35%) | 0/58 | 5/45 (11.11%) | 0/45 |
| **TB Symptoms** | 63/63 (100%) | 0/58 | 45/45 (100%) | 0/45 |
| **Laboratory findings** |  |  |  |  |
| WBC ^a^ count, (10^9^ cells/L) | 7.68 (5.24-8.44) | 6.55 (5.81-7.94) | 6.91 (4.87-8.27) | 6.07 (5.36-7.91) |
| Lymphocyte count, (10^9^ cells/L) | 1.58 (0.91–1.96) | 2.12 (1.91-2.77) | 1.24 (0.81–1.65) | 2.24 (1.85-2.31) |
| Lymphocytopenia (<500/µL) | 5/63 (7.94%) | 0/58 | 5/45 (11.11%) | 0/45 |
| Thrombocytopenia(<150 × 10^3^/µL) | 4/63 (6.35%) | 2/58(3.45%) | 4/45 (8.89%) | 2/45 (4.44%) |
| **HIV infection ^b^** | 3/63 (4.76%) | 0/58 | 3/45(6.67%) | 0/45 |
| **Positive sputum acid-fast bacilli** | 22/63 (34.92%) | n.a. ^d^ | 25/45 (55.56%) | n.a. ^d^ |
| **Positive culture** | 63/63 (100.00%) | n.a. ^d^ | 45/45(100.00%) | n.a. ^d^ |
| **Positive IGRA ^c^** | 56/63 (88.89%) | n.a. ^d^ | 41/45 (91.11%) | n.a. ^d^ |
| **CXR suggesting TB** | 38/63 (60.32%) | 0/58 | 31/45 (68.89%) | 0/45 |

Age (years): mean ± SD; other data are presented as median (inter-quantile range).

The age and gender showed no statistical differences between healthy control (HC) group and TB group (compared using Mann-Whitney U test or chi-square test, *P* < 0.05).

CXR：Chest X-ray.

“a”, WBC: white blood cell; “b”, HIV: human immunodeficiency virus; “c”, IGRA: IFN-γ release assay (T-SPOT.TB); “d”, n.a.: not available.
